# Micro-Power Harvesting from Electromagnetic Interferences in Power Systems

**DOI:** 10.3390/mi17091056

**Published:** 2026-09-03

**Authors:** Moreno d’Ambrosio, Gabriele Marasca, Massimo Calvo, Aldo Romani, Carmelo Corsaro, Salvatore Patanè

**Affiliations:** 1 Department of Mathematical and Computer Sciences, Physical Sciences and Earth Sciences (MIFT), University of Messina, 98166 Messina, Italy; moreno.dambrosio@studenti.unime.it (M.d.); massimo.calvo@unime.it (M.C.); carmelo.corsaro@unime.it (C.C.); 2 Advanced Research Center on Electronic Systems “Ercole De Castro”, University of Bologna, 47522 Cesena, Italy; gabriele.marasca2@unibo.it (G.M.); aldo.romani@unibo.it (A.R.); 3 Department of Electrical, Electronic, and Information Engineering “Guglielmo Marconi” (DEI), University of Bologna, 47522 Cesena, Italy

**Keywords:** electromagnetic interference, energy harvesting, radio frequency, ultra-low-power system

## Abstract

The increasing demand for real-time monitoring systems has accelerated the adoption of distributed sensors operating in the ultra-low-power regime. However, in retrofittable and hard-to-access applications, providing a continuous external power supply is challenging, while batteries are limited by their lifetime and maintenance requirements. Energy harvesting represents a promising approach to enable autonomous sensor operation by exploiting ambient energy sources, including photovoltaic, thermal, mechanical, and electromagnetic sources. In this work, different circuit topologies for near-field electromagnetic energy harvesting are investigated through simulation and experimental validation. The proposed approach exploits the electromagnetic interference generated by shielded commercial power electronics, such as drivers and power supplies, as an available energy source. The results demonstrate the capability of compact and easily deployable circuits to capture and convert near-field electromagnetic energy into usable electrical power. The proposed methodology provides a flexible solution for powering low-power monitoring systems and can be adapted to different environments by tailoring the harvesting circuit parameters to the available electromagnetic source.

## 1. Introduction

Energy harvesting is a promising technology to support the transition toward sustainable and low-maintenance power systems. In the broader framework of decarbonization and electrification, increasing attention has been devoted to deploying distributed sensing infrastructures capable of improving monitoring, efficiency, and reliability of energy systems. Applications range from smart grids and renewable generation plants to industrial automation and predictive maintenance platforms [1]. In this context, the rapid expansion of the Internet of Things (IoT) has created a growing demand for autonomous sensing nodes operating with ultra-low power consumption. A critical bottleneck in large-scale deployment remains the power supply of distributed sensors, particularly in retrofit scenarios where wired power is unavailable and periodic battery replacement is economically or logistically impractical [2]. Consequently, energy harvesting holds the promise of maintenance-free sensing systems. Conventional energy-harvesting approaches typically exploit well-characterized sources such as photovoltaic energy [3], thermal gradients through thermoelectric generators [4], mechanical vibrations via piezoelectric or electromagnetic transducers [5,6], and ambient radio-frequency (RF) signals from communication infrastructures including Wi-Fi, cellular networks, and broadcast transmissions [7]. Conducted energy harvesting from power lines has also been widely investigated for grid monitoring applications [8,9]. Thermoelectric harvesters have also been proposed to extract power from waste heat in grid transformers [10]. However, while these sources are relatively predictable, they require access and installations in complex infrastructures, following strict regulatory frameworks.

In this field, considerably less attention has been devoted to harvesting energy from unintended and poorly characterized sources such as electromagnetic interference (EMI) generated by electronic power converters. Traditionally regarded as an undesirable by-product and mitigated to comply with electromagnetic compatibility (EMC) regulations, EMI cannot be completely suppressed. Power converters inherently generate time-varying electromagnetic fields through high-frequency switching based on pulse-width modulation (PWM) or space vector modulation (SVM) [11,12]. Although difficult to characterize spectrally and spatially, these emissions represent a ubiquitous electromagnetic energy source that could power ultra-low-power devices monitoring the converter or its surrounding environment. This opportunity is increasingly relevant as modern energy systems extensively employ power electronic converters, including photovoltaic inverters, DC microgrid converters, motor drives, and other switching-based systems. Moreover, wide-bandgap semiconductors such as silicon carbide (SiC) and gallium nitride (GaN) enable higher switching frequencies and power densities while intensifying electromagnetic emissions [13]. Consequently, electromagnetic fields from several tens of kilohertz to a few megahertz are increasingly common in industrial and domestic environments, providing a continuously available local energy source near operating converters.

However, exploiting EMI for energy harvesting remains challenging because converter-generated fields strongly depend on topology, circuit layout, operating conditions, and component orientation, resulting in spatial non-uniformity and temporal variability. Their broadband spectra may shift with operating conditions, while field amplitudes are relatively low, especially below 30 MHz. Metallic enclosures can further suppress far-field radiation, making magnetic near-field coupling the dominant transfer mechanism. These characteristics complicate both receiver design and power-conditioning optimization. Accordingly, only a limited number of studies have investigated energy harvesting from electromagnetic noise generated by power converters [14,15]. Most previous research focuses on RF harvesting at GHz frequencies [7,16,17], leaving the sub-1 MHz region largely unexplored. Here, energy is predominantly captured through inductive receivers operating in the quasi-static near-field regime, whose design principles differ substantially from conventional RF rectennas [18], although analogies exist with near-field communication (NFC) and radio-frequency identification (RFID) systems. The variable spectral content of converter-generated EMI introduces a key trade-off between resonant amplification and bandwidth. High-Q resonant structures increase induced voltage but are sensitive to switching-frequency fluctuations and component tolerances [19], whereas broadband receivers offer greater robustness at the cost of lower voltage gain, potentially preventing reliable startup of ultra-low-power rectification and DC–DC conversion stages. This trade-off is critical because commercial ultra-low-power boost converters typically require minimum input voltages of about 0.3–0.4 V and input currents of several tens of microamperes [20,21,22,23,24]. Although energy harvesting from electromagnetic interference and from stray magnetic fields has been previously investigated, the harvesting of low-frequency EMI radiated by power electronic converters through predominantly magnetic near-field coupling remains a comparatively unexplored scenario.

This paper presents an experimental investigation of low-frequency radiated EMI harvesting from a commercial switching power supply focusing on a ferrite-based resonant receiving structure operating in near-field regime and evaluates four rectifier topologies employing different device technologies and impedance-matching networks. The receiver is intentionally positioned in the near-field region of the converter, where the energy transfer is predominantly governed by magnetic coupling rather than by far-field electromagnetic radiation. This operating condition differs substantially from the RF energy-harvesting approaches commonly reported in the literature, which generally address far-field radiation and higher-frequency signals. The results demonstrate that low-frequency radiated EMI can be exploited as a practical energy source, opening new perspectives to introduce battery-less micro-power retrofittable monitoring systems in power systems or power electronics environments.

The structure of this article is divided as follows: first in Section 2 we measure and discuss EMI sources and their changes from one to the other. Then, in Section 3 we describe the transducer and four possible topologies to rectify the EMI source. In Section 4 we present the simulation for every topology firstly with an optimized matching network for every rectifier and then with the same matching network for every topology. In Section 5 the experimental results are provided and discussed. Finally, in Section 6 the conclusions of this work are reported.

## 2. General Approach

**Figure 1:**
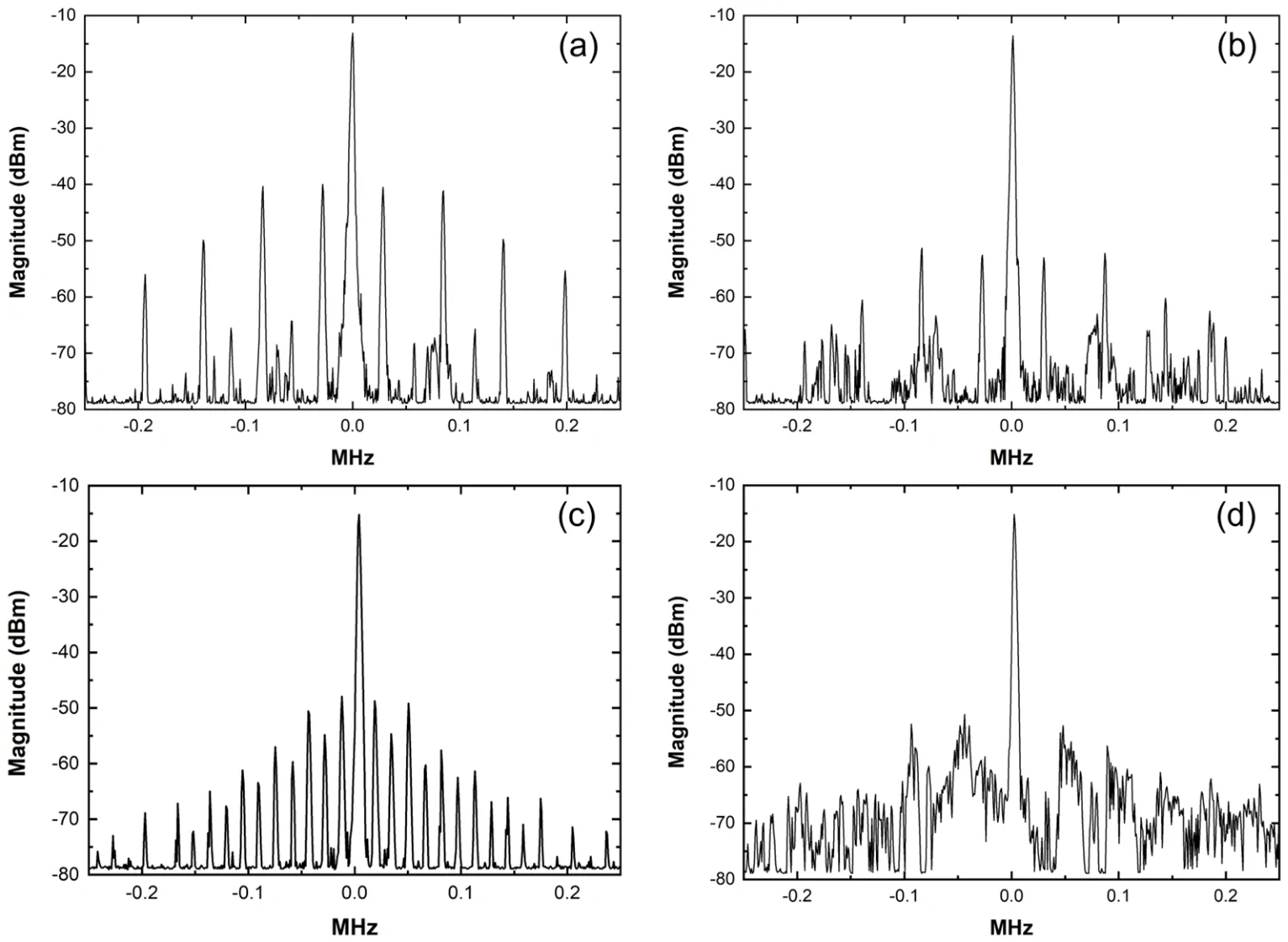
Near-field emission spectra of (**a**,**b**) commercial LED strip power supplies and (**c**,**d**) commercial laptop power supplies, acquired with a resolution bandwidth of 1 kHz while supplying a 50 Ω resistive load.

Switched-mode power supplies typically operate using specific switching modulation techniques (e.g., pulse-width modulation (PWM)) operating in the range of 50–200 kHz, generating electromagnetic interference (EMI) associated with high transient currents and gate driving of power transistors. Although mitigation of these emissions through filtering and shielding has been extensively addressed in the electromagnetic compatibility (EMC) literature [25,26], their spectral content is inherently broad, with radiated components and harmonics extending up to several MHz. While most studies focus on suppressing electromagnetic emissions, the present work focuses on harvesting electromagnetic energy radiated by power supplies in their close proximity and within the low- and medium-frequency spectrum, below 1 MHz. Figure 1 reports examples of near-field emission spectra measured in proximity of different types of commercially available power supplies. The spectra were acquired using an Advantest R3141B spectrum analyzer (Advantest Corporation, Tokyo, Japan), interfaced to a computer via the IEEE-488 (GPIB) port for data acquisition, and wired via a coaxial cable to an inductor (0.6 mH) working as a near-field probe for EMI signal pickup. Measurements were performed at a fixed probe position and distance (≈1 cm) from the metallic shield to ensure consistency across experiments.

Figure 1 reports the EMI spectra measured from four commercially available power supplies, namely two low-cost constant-voltage LED strip drivers (Figure 1a,b) and two laptop power supplies (Figure 1c,d). The LED drivers exhibit multiple discrete spectral components, which can be attributed to the switching activity and its associated harmonics, together with a comparatively pronounced broadband noise floor. The laptop power supplies, in contrast, exhibit a more structured spectral distribution, characterized by a dominant switching component and its harmonics, as well as a lower broadband noise floor. These differences may reflect the different converter architectures and the more stringent electromagnetic compatibility requirements typically applied to laptop power supplies. Among the investigated sources, the power supply characterized by the spectrum in Figure 1a was selected as the EMI source for all subsequent measurements and energy-harvesting experiments. It provides a nominal output of 5 V up to 30 A, corresponding to a maximum rated power of 150 W. During the experiments, a 2.2 Ω power resistor was used as the load.

Since near-field measurements are strongly dependent on the probe position and distance, the reported results should be interpreted as clear evidence of the presence of EMI and its spectral distribution, rather than as a quantitative characterization. The intensity of the EMI field decreases by many decibels at the second harmonic. Hence, circuit design should primarily target the fundamental switching frequency, which is inherently unstable due to the intrinsic operating principles of these systems. This non-negligible detail introduces the first major design challenge: the need to realize a circuit with a response bandwidth as wide as possible. This requirement is not easily satisfied, since conventional resonant circuits operating in the hundreds of kHz range typically exhibit very narrow bandwidths, with quality factors in the order of several hundred, making them unsuitable for the intended purpose. Conversely, designing a resonant circuit with a low-quality factor requires the introduction of dissipative components, which directly conflicts with the need to maximize energy conversion efficiency. In other words, the design must strike a compromise between bandwidth and conversion efficiency. Within this framework, an initial prototype was implemented using a simple LC resonant circuit, consisting of a ferrite-core inductor and a low-loss capacitor. In the proposed idea, the ferrite-based antenna acts as the transducer responsible for capturing the ambient electromagnetic energy, which is subsequently converted into a usable DC voltage by a rectifier circuit. The following sections describe the individual stages of this energy-harvesting process, discuss the experimental results, and outline potential applications of the proposed approach.

## 3. System Design

A ferrite coil of relatively small dimensions (50 × 8 × 5 mm) has been selected as the transducer to collect energy from the time-varying concatenated electromagnetic fields. The ferrite dimensions were selected as a practical compromise between magnetic harvesting performance, commercial availability, and integration constraints, with particular consideration given to the intended use of the transducer in compact self-powered sensor and IoT nodes; larger ferrite cores, although potentially beneficial for magnetic coupling, would compromise the compactness and practical integration of the receiving system. Moreover, the unpredictable distribution of electronic components within the source motivates the use of a ferrite with a lateral size comparable to the centimeter-scale extent of the near-field region. It is worth noting that energy harvesters employed to power retrofittable sensors in smart grids and data centers often exhibit dimensions ranging from a few centimeters to a few tens of centimeters [27,28].

The low-frequency energy-harvesting antenna has been realized using a MnZn ferrite magnetic rod with a compact winding of 120 turns. [29,30]. MnZn ferrite is well suited to this application because of its relatively high initial permeability µ_r_ (typically in the range 100–2000), low losses at low frequencies, and suitability from a few tens of hertz up to approximately 1.5–2 MHz. The winding is tightly packed along the rod to maximize magnetic coupling and reduce flux leakage. The winding has a measured inductance of approximately 650 µH and a series resistance of approximately 20 Ω at 1 kHz. The relatively high DC resistance represents a significant source of loss for an energy-harvesting application and has been explicitly considered in the receiver design.

**Figure 2:**
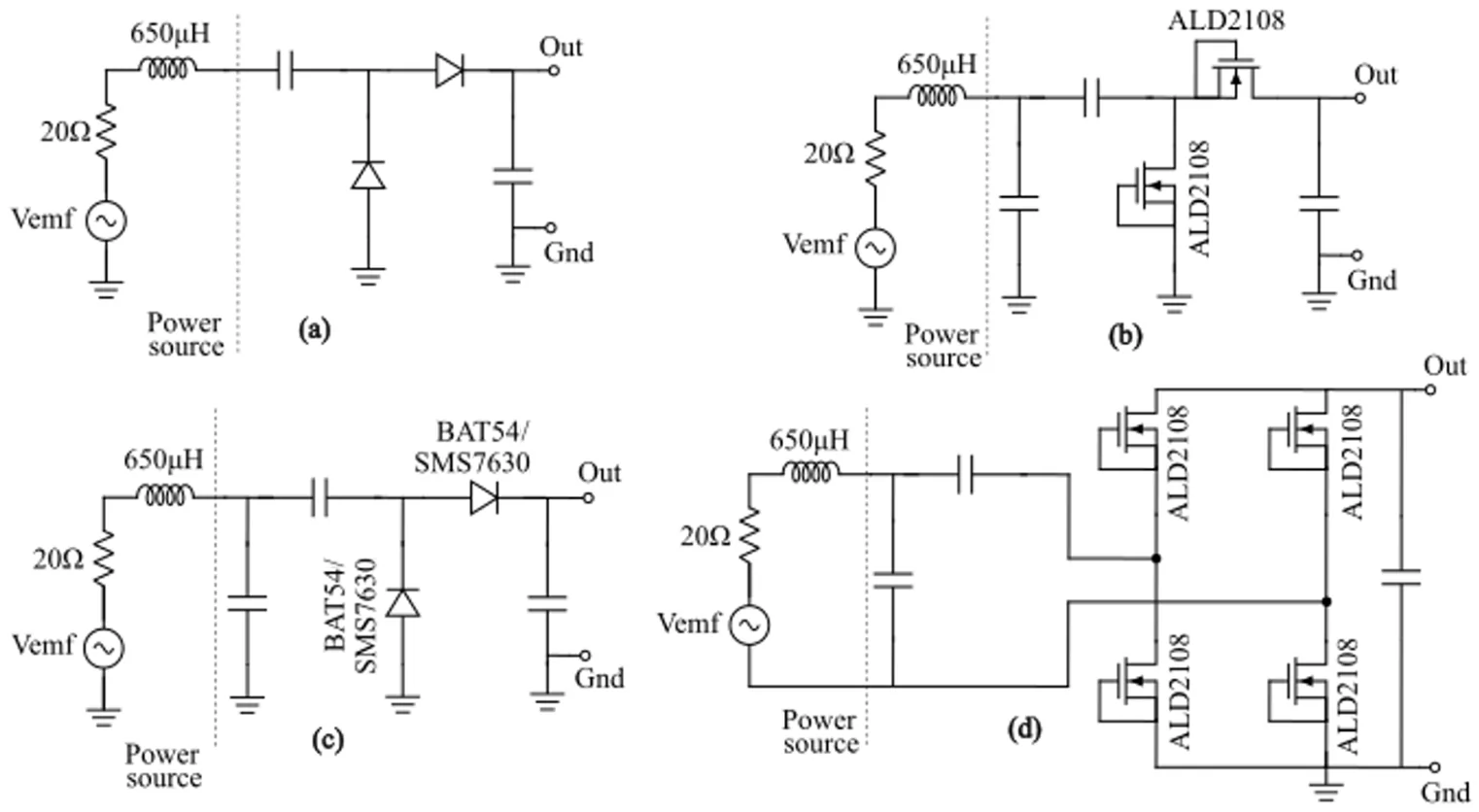
Schematics of the four considered rectifier configurations realized for EMI energy harvesting: (**a**) ideal matching with a series capacitor and a voltage doubler; (**b**,**c**) matching network with parallel capacitor and voltage doubler rectifier based on zero-threshold MOSFETs and Schottky diodes; (**d**) matching network with parallel capacitor and a full-bridge rectifier.

A first approach for maximum power transfer could consist of suppressing the reactance produced by the inductor by adding a series capacitance (Figure 2a). Using Equation (1), it is possible to calculate the required capacitance value at a given frequency f_0_:

(1) $$X_{L}=\;2\pi f_{0}L\;=\;-\;X_{C}\;=\;\frac{1}{2\pi f_{0}C}.$$

However, it is important to also consider the effect of the rectifier in this evaluation due to its intrinsic impedance. The main drawback of this approach is the difficulty in harvesting low-power ambient sources. This is due to the low source resistance on the order of 20 Ω compared to the high input resistance presented by the rectifier.

**Figure 3:**
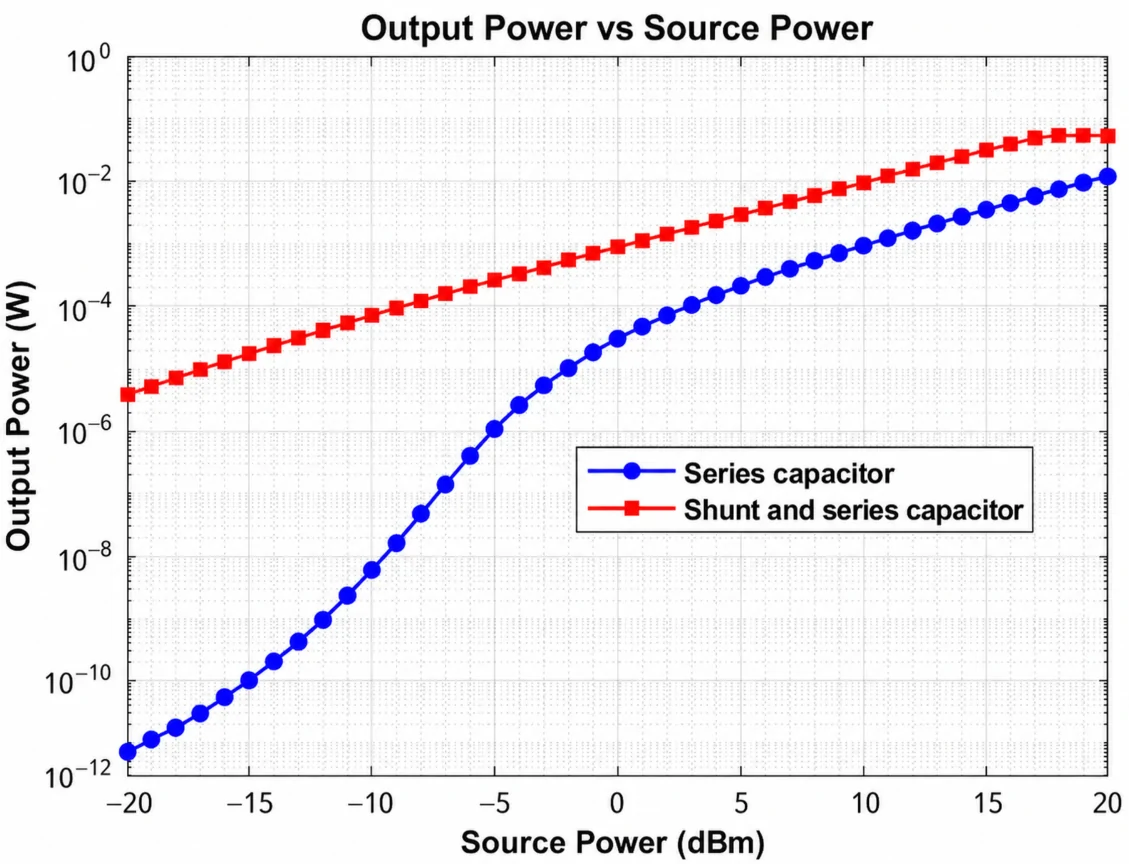
Rectified output power over source power for the topology with a series capacitor (Figure 2a) and the topology with shunt and a series capacitor (Figure 2c) to match the Greinacher voltage doubler using BAT54 as diodes.

To overcome this mismatch, we adopted the topology proposed in Figure 2c, which is capable of modifying the real part of the circuit impedance seen by the source bringing it closer to the source resistance. Figure 3 illustrates the rectified output power over source power for Figure 2a,c topology. It is worth noting that, under the investigated low-frequency and low-voltage operating conditions, the rectifier performance is not determined solely by the circuit topology. The choice of semiconductor technology and device also plays a significant role, as parameters such as conduction losses, threshold or forward voltage, leakage current, parasitic capacitances, and switching characteristics can substantially affect the conversion efficiency at the very low power levels available from the EMI source. For these simulations, BAT54 diodes for the Greinacher voltage doubler were used. In both scenarios, the capacitor values were optimized via harmonic balance simulations to extract the maximum amount of power. It can be observed that, for every input power considered, it is better to use the topology with shunt and series capacitors compared to the series capacitor configuration.

Those results are obtained with a load resistance of 17 kΩ for the topology with the shunt and series capacitors and of 1.2 kΩ for the series capacitor configuration, which are the resistance values that maximize the output power with an input power of 0 dBm at the source. It is also worth mentioning that for higher levels of input power, the series capacitance topology can achieve higher output power compared to the shunt and series capacitor configuration. The choice of the most suitable topology for rectifying the harvested signal is not straightforward, since the two configurations under consideration (Greinacher voltage doubler and full-wave rectifier) exhibit significantly different characteristics [15]. In particular, the Greinacher doubler provides higher output voltages and lower currents, whereas the full-wave rectifier offers lower output resistance and higher currents, but lower output voltages. These two output characteristics reflect a fundamental trade-off between output voltage and current delivery capability. In practice, the preferred configuration would be the one combining high output voltage with low output resistance, as this approaches the behavior of an ideal voltage source. Moreover, sufficiently high voltage levels are required to satisfy the activation threshold of a step-up stage. Typically, output voltages of at least 0.35–0.4 V with currents of a few tens of μA can be considered a target, as these are suitable to drive a low-power step-up circuit [31,32]. Another critical aspect concerns the choice of rectifying device and circuit topology, which are critical design parameters in low-power energy-harvesting systems, as they directly affect start-up behavior, conversion efficiency, and the minimum harvestable input power. Although both Schottky diodes and ultra-low-threshold (zero-threshold) MOSFETs have been widely adopted for rectification in energy-harvesting applications, their performance strongly depends on the operating conditions and the characteristics of the available electromagnetic source [33,34,35]. The highly variable amplitude and low power density of radiated EMI in the sub-30 MHz range may favor specific rectification devices and circuit topologies over others. To test this hypothesis, four rectifier prototypes were designed and experimentally evaluated. All prototypes share an identical energy-harvesting front-end and differ exclusively in the rectifying devices and topology. The investigated configurations are shown in Figure 2 and consist of three Greinacher voltage doublers based on ALD2108 (Advanced Linear Devices, Inc., Sunnyvale, CA, USA) MOSFETs (Figure 2b), BAT54C (Diodes Incorporated, Plano, TX, USA) and SMS7630 (Skyworks Solutions, Inc., Irvine, CA, USA) Schottky diodes (Figure 2c), and a full-bridge rectifier employing ALD2108 MOSFETs (Figure 2d).

## 4. Simulations

For the simulations, we considered a source capable of providing a maximum power of −10 dBm to the load at a frequency of 100 kHz with a source resistance R_S_ = 20 Ω, corresponding to a sinusoidal input open-circuit voltage of amplitude V_0_ = 0.1265 V. These are representative values typically measured in proximity to the considered power supplies. These values are derived from the well-known Equation (2), considering a sinusoidal voltage and calculating the load power as:

(2) $$P\;=\;\frac{V_{0}^{2}}{8R_{S}}.$$

It is worth mentioning that the harvested power also depends on the coil’s position on the power converter case, making it difficult in real conditions to provide a precise expected value for a given source.

Based on these parameters, the optimum capacitance and resistance values for the matching network of the aforementioned topology were evaluated and are summarized in Table 1. These values were obtained using harmonic balance simulations on Advanced Design System 2026 1.0 with 16 harmonics, representing a reasonable trade-off between accuracy and simulation time. Regarding the capacitors, ideal components were utilized.

**Figure 4:**
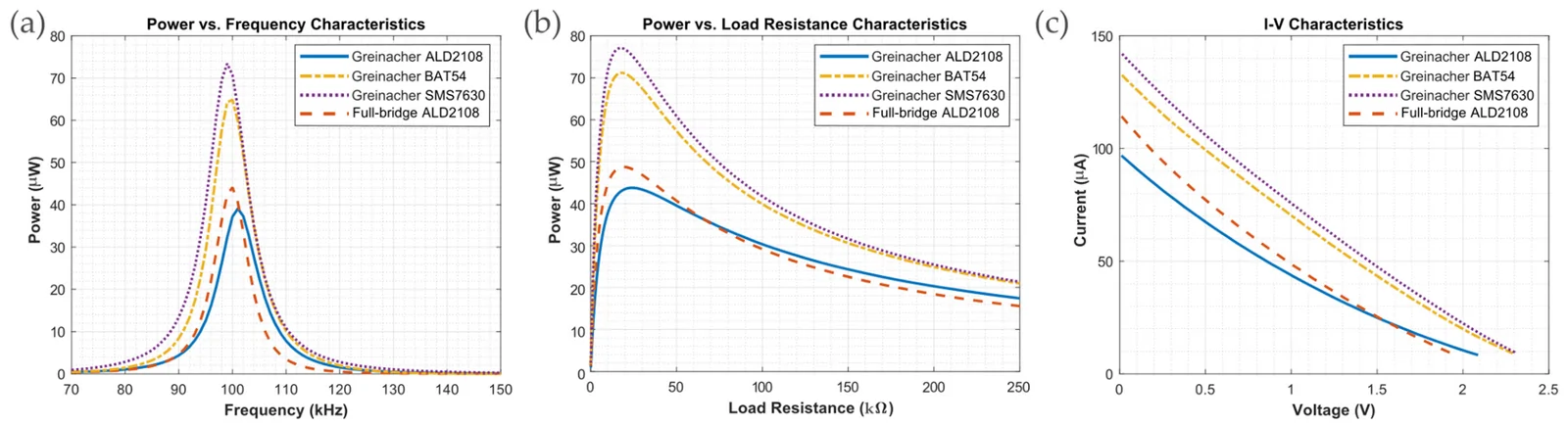
Simulations of (**a**) the DC output power delivered to the optimum loads in Table 1 versus frequency, (**b**) the DC output power versus load resistance at 100 kHz and (**c**) the I-V static transfer characteristics at 100 kHz for the different rectifier topologies in Figure 2b–d. Blue solid, yellow dash-dotted and purple dotted curves correspond to Greinacher configurations with ALD2108 MOSFETs, BAT54 diodes, and SMS7630 diodes, respectively. Red dashed curves correspond to full-bridge configuration with ALD2108 MOSFETs.

Values in Table 1 have been employed to simulate the primary performance metrics of those circuits. The corresponding simulation results are reported in Figure 4. The frequency responses of the different circuits, showing the rectified DC power on the optimum loads across a frequency range from 70 to 150 kHz with steps of 1 kHz, is reported in Figure 4a.

In Figure 4b the rectified DC power of the different circuits is reported with different load resistance values with steps of 1 kΩ at a fixed frequency of 100 kHz, while the I-V static characteristics of the different circuits at a frequency of 100 kHz are shown in Figure 4c. For this figure, the voltage steps were obtained by sweeping the load resistance from 100 Ω to 250 kΩ in 1 kΩ increments. The comparison between the three simulated plots in Figure 4 consistently suggests the Greinacher configuration realized using the SMS7630 diodes as the most promising for the considered application.

**Figure 5:**
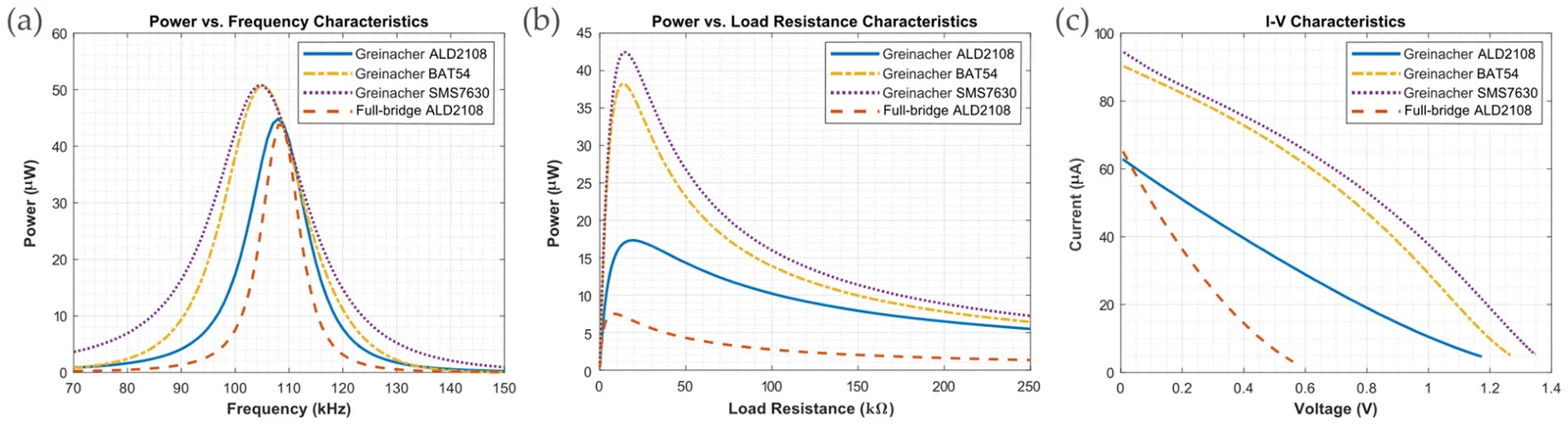
Simulations of (**a**) the DC output power versus frequency, (**b**) the DC output power versus load resistance at 100 kHz and (**c**) the I-V static transfer characteristics at 100 kHz using a series capacitor of 4.7 nF and a shunt capacitor of 3.6 nF for the different rectifier topologies in Figure 2b–d. Blue solid, yellow dash-dotted and purple dotted curves correspond to Greinacher configurations with ALD2108 MOSFETs, BAT54 diodes and SMS7630 diodes, respectively. Red dashed curves correspond to full-bridge configuration with ALD2108 MOSFETs.

It is worth mentioning that at different power levels we expect different performance from the various topologies. For example, with an input power of 2 dBm, the BAT54 topology outperforms the SMS7630. This is due to the lower I_S_ parameter of the BAT54 compared to the SMS7630. However, this power level is significantly higher than what can realistically be extracted from the measured commercial power supply. Although evaluating the ALD2108 topologies was reasonable to compare zero-threshold MOSFETs with Schottky diodes, they are ultimately outperformed by the diode topologies at these operating power levels. To validate the simulations against real-world measurements, since the exact source parameters cannot be known a priori due to the variable behaviour of the power converter, we opted to use the same matching network across all topologies. The source input power used in simulations was −10 dBm, with a series capacitor of 4.7 nF and a shunt capacitor of 3.6 nF. Figure 5a illustrates the power versus frequency curves obtained using steps of 1 kHz with the values of load resistance of 19 kΩ for the transistor-based voltage doubler with ALD2108, 14 kΩ for the voltage doubler topology with BAT54 diodes, 15 kΩ for the voltage doubler topology with SMS7630 diodes, and 9 kΩ for the full-bridge rectifier with ALD2108 transistors. Figure 5b depicts the power versus load resistance with steps of 1 kΩ at a fixed reference frequency of 100 kHz, while Figure 5c depicts the I-V characteristics at 100 kHz, sweeping the load resistance from 100 Ω to 250 kΩ in 1 kΩ increments. As in previous simulations, the comparison between the three plots suggests that the voltage doubler with SMS7630 diodes is the best candidate in the considered scenario.

## 5. Experimental Results

**Figure 6:**
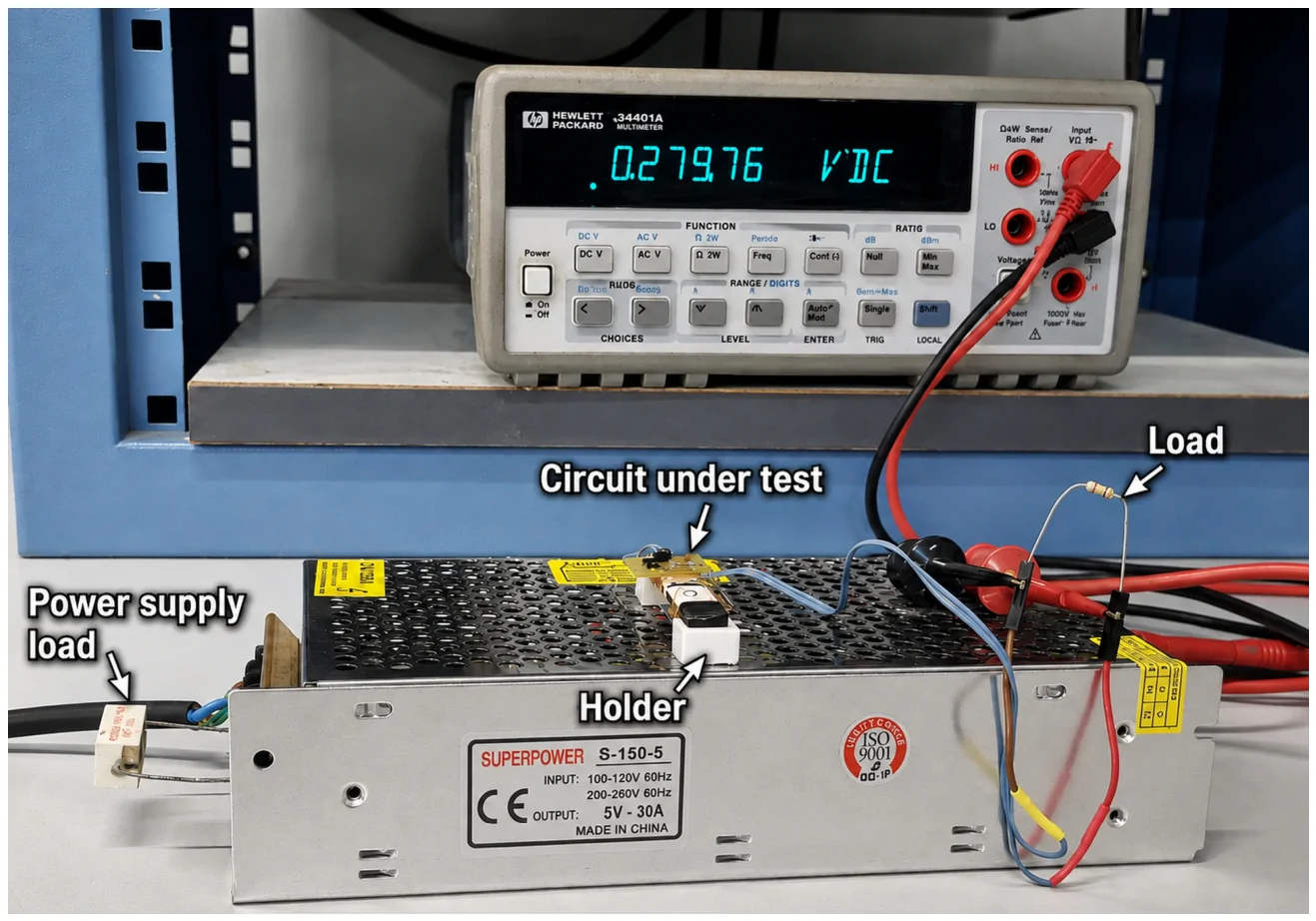
Photograph of the experimental measurement setup.

The four simulated rectifying devices were realized and tested using the aforementioned antenna as power source. The experimental setup is shown in Figure 6. It comprises a commercial LED strip power supply, employed as an intentional EMI source, and the circuit under test, which is positioned above its enclosure and mechanically fixed to a dedicated holder to ensure repeatable positioning and measurement conditions. In all experiments, the antenna is maintained at a constant distance of approximately 1 cm from the upper surface of the power supply, thereby providing a well-defined and reproducible electromagnetic coupling configuration. The obtained energy is supplied to a resistive load and the voltage developed across this load is measured using a Keysight/Agilent 34401A digital multimeter (Agilent Technologies, Inc., Loveland, CO, USA). The power supply is operated under a controlled electrical load provided by the high-power resistor shown on the left, ensuring reproducible operating conditions during the experiments.

From an implementation perspective, we used SMD components, while further integration into a dedicated IC could provide additional size reduction. However, the overall dimensions of the proposed harvester are primarily constrained by the ferrite-based receiving transducer, which is required to provide sufficient magnetic coupling at the investigated sub 100 kHz frequencies. Therefore, further miniaturization of the electronic circuitry alone would have a limited impact on the overall system size. Moreover, the typical footprint of microprocessors used in retrofittable sensor platforms is generally of the order of a few cm^2^ [36], further limiting the system miniaturization.

**Figure 7:**
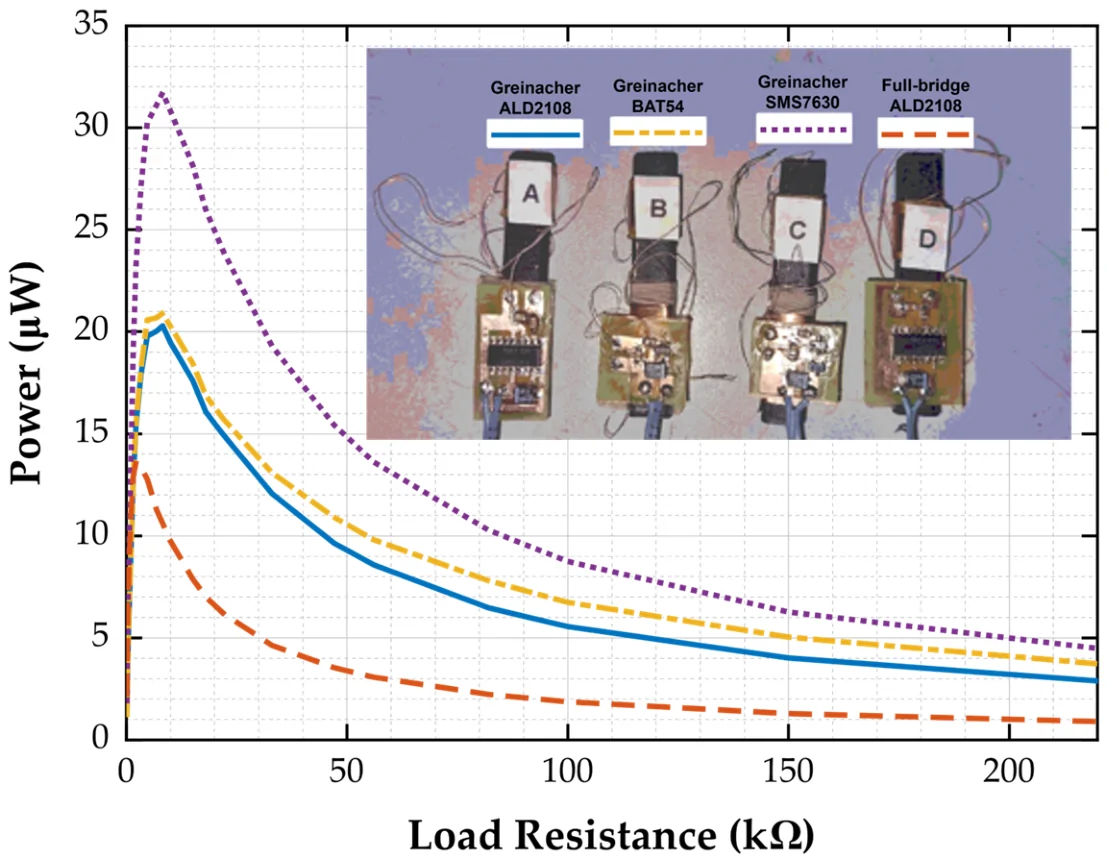
Measured output power as a function of the load resistance for the four EMI harvesting prototypes. Inset shows the corresponding ferrite antenna prototypes, labeled A, B, C, and D, corresponding to Greinacher rectifiers with (A) ALD2108 MOSFETs, (B) BAT54 diodes, (C) SMS7630 diodes and (D) full-bridge configuration with ALD2108 MOSFETs.

**Figure 8:**
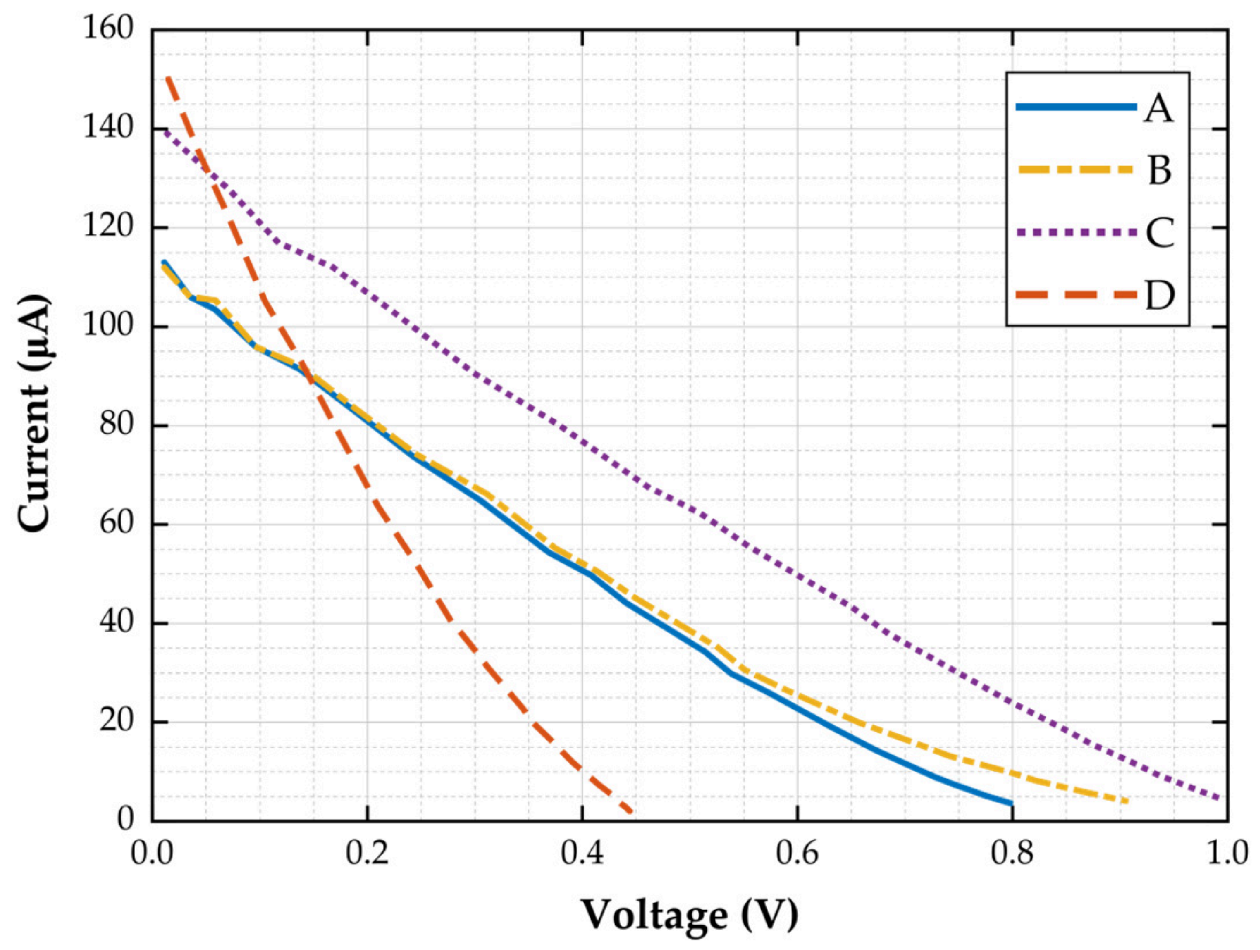
Measured current–voltage (I–V) characteristics of the four EMI energy-harvesting prototypes under identical excitation conditions. Labels A–D correspond to Greinacher rectifiers with (A) ALD2108 MOSFETs, (B) BAT54 diodes, (C) SMS7630 diodes and (D) full-bridge configuration with ALD2108 MOSFETs.

All these devices were tested using a simple commercial LED strip power supply as the EMI source, whose emission spectrum is shown in Figure 1a. To measure the power delivered by the devices (P_L_), the output voltage V_L_ of the rectifier was measured as a function of the load resistance R_L_. In this way, for each measurement, it was possible to calculate the static power transfer characteristics and the I-V curves:

$$I_{L}\;=\frac{V_{L}}{R_{L}}\;P_{L}\;=\frac{V_{L}^{2}}{R_{L}},$$

where I_L_ is the current that flows in the load resistance. In particular, power curves (Figure 7) allow the maximum power point (MPP) to be identified, while the I–V characteristics (Figure 8) are used to determine the most suitable device for the intended purpose, considering also the requirements in terms of operating voltage of power conversion circuits and their input currents.

From Figure 7, it can be immediately observed that device C, which uses SMS7630 diodes in a Greinacher configuration, is capable of delivering the highest amount of power. The MPP is obtained with a load resistance of 8 kΩ and corresponds to approximately 32 µW. It is worth noting that the device implemented in a full-bridge configuration, although delivering a lower power (slightly less than half), exhibits a much lower output impedance of approximately 2 kΩ. This result was somewhat expected, since the full-bridge rectifier is capable of providing higher currents than the Greinacher configuration if the proper load is employed. The other two configurations (device A and device B) provide intermediate results and are therefore of limited interest.

The I-V curves (Figure 8) confirm the above observations; in particular, configuration C provides an open-circuit voltage of about 1 V and a short-circuit current of about 140 µA. It can be observed that this configuration delivers currents of approximately 80 µA at voltages of about 0.4 V, indicating the presence of an operating point compatible with voltage and current requirements needed to drive a low-power step-up circuit. The measurements align with the general trends predicted by the simulations, with a degradation in the simulated performance of the ALD2108 topologies caused by the frequency shift reported in Figure 5a, that can occur due to parasitic elements that could not be accurately embedded in the SPICE model of the component. Furthermore, the I-V curves make it possible to predict the maximum power point without directly measuring the output power. According to the maximum power transfer theorem, in case of matched reactance, the power delivered to the load is maximum when the load resistance is equal to the output resistance of the Thévenin source (R_out_)

$$R_{L}\approx {\;R}_{\mathrm{out}}.$$

The value of R_out_ can directly be extracted by the linear approximation of the I-V characteristics, which in the present case is about 7.2 kΩ. This insight is crucial for correctly designing the step-up circuit. Overall, the I-V analysis provides a clear physical interpretation of the measured power curves and highlights the intrinsic trade-off between output voltage and current imposed by the rectifier topology and device technology. This must be evaluated carefully, since any output circuitry will draw some current and affect the voltage, which must remain above the minimum allowed value for operation. Based on the combined analysis of power curves and I-V characteristics, configuration C, employing SMS7630 Schottky diodes in a Greinacher topology, was selected as the optimal solution. Although the full-bridge configuration exhibits a lower output impedance and higher short-circuit current, its reduced open-circuit voltage would prevent the reliable activation of a commercial power management integrated circuit (PMIC). Typical values for cold starting state-of-the-art PMICs are voltages of at least 300 mV with currents drawn from the source on the order of 10 µA. Hence, since configuration C provides both an output voltage of approximately 0.4 V and currents in the order of several tens of microamperes, the minimum start-up requirements of a PMIC can be considered as satisfied with a certain safety margin. These results indicate that SMS7630 diodes in the voltage doubler configuration represent the best compromise between output voltage, current capability, and practical usability within the proposed energy-harvesting system.

## 6. Conclusions

This work has presented an experimental investigation of low-frequency electro-magnetic interference (EMI) harvesting from commercially available switching power supplies. Unlike conventional RF energy-harvesting approaches based on dedicated transmitters, and far-field electromagnetic propagation, the proposed method exploits unintended electromagnetic emissions as a locally available energy source in real operating environments. In particular, the focus is on the exploitation of the localized magnetic near field generated by the switching activity of power electronic converters, enabling energy harvesting at a very short distance from the EMI source. A ferrite-based resonant receiving structure has been realized to operate in the sub-MHz range, with near-field magnetic coupling. The receiver is positioned at a controlled distance of approximately 1 cm from the power-supply surface, providing a well-defined and repeatable near-field coupling condition. The analysis highlighted the fundamental trade-off between bandwidth and voltage amplification imposed by the variability of switching frequencies. To address this aspect, a moderately low-Q resonant network has been designed in order to ensure sufficient robustness against spectral fluctuations of the EMI source. The proposed approach therefore combines low-frequency EMI harvesting with near-field magnetic coupling and resonant energy conversion, enabling the local recovery of energy from EMI emissions of power electronic converters. Four rectifier topologies based on different device technologies have been experimentally evaluated under identical conditions. The results demonstrate that the voltage doubler configuration employing SMS7630 Schottky diodes provides the best overall performance, achieving a maximum harvested power of approximately 32 µW with an optimal load of about 8 kΩ. The corresponding voltage and current levels are compatible with the start-up requirements of low-power DC–DC converters, confirming the practical feasibility of the proposed approach. Although the harvested power remains limited, the study demonstrates that low-frequency magnetic near-field EMI can be effectively exploited to power micro-watt-level sensing systems without the need for dedicated energy sources. As discussed previously, the generated voltages and currents are sufficient to cold start several PMICs available on the market, which can accumulate energy locally on capacitors or rechargeable batteries. The estimated available power is also compatible with sustaining the typical leakage currents of capacitors or supercapacitors, which can reach values of up to several µA. A positive power budget is then expected, and even complex sensing tasks can be performed with an appropriate duty cycle. This opens new perspectives for battery-less retrofit applications in power electronics environments, where electromagnetic emissions are inherently present and can be locally harvested through near-field magnetic coupling. Future work will focus on improving the overall system efficiency through optimized impedance matching and adaptive tuning techniques, as well as on the integration of the harvesting stage with ultra-low-power power management circuits for fully autonomous sensing platforms.

## Figures and Tables

**Table 1:** Tested configurations for rectification and impedance matching.

Rectifying Device	Series Capacitance	Shunt Capacitance	Load Resistance
ALD2108 Single ended	400 pF	3.6 nF	24 kΩ
BAT54 Diode	550 pF	3.6 nF	18 kΩ
SMS7630 Diode	550 pF	3.6 nF	17 kΩ
ALD2108 Fully differential	51 nF	3.9 nF	19 kΩ

## Data Availability

The original contributions presented in this study are included in the article. Further inquiries can be directed to the corresponding author.

## Abbreviations

The following abbreviations are used in this manuscript:

RF	Radio-frequency
DC	Direct current
EMI	Electromagnetic interference
LED	Light-emitting diode
IC	Integrated circuit
IoT	Internet of things
MOSFET	Metal-oxide-semiconductor field-effect transistor
MPP	Maximum power point
NFC	Near-field communication
PCB	Printed circuit board
PMIC	Power management integrated circuit
PWM	Pulse-width modulation
RFID	Radio-frequency identification
SMD	Surface mount device
SVM	Space vector modulation
